# *Salmonella enterica* in Pinnipeds, Chile

**DOI:** 10.3201/eid1712.111103

**Published:** 2011-12

**Authors:** Natalie Sturm, Pedro Abalos, Alda Fernandez, Guillermo Rodriguez, Pilar Oviedo, Viviana Arroyo, Patricio Retamal

**Affiliations:** Universidad de Chile, Santiago, Chile (N. Sturm, P. Abalos, P. Oviedo, V. Arroyo, P. Retamal);; Instituto de Salud Pública, Santiago (A. Fernandez);; Buin Marino, Santiago (G. Rodriguez)

**Keywords:** pinnipeds, marine mammals, Salmonella enterica, Chilean coast, Chile, zoonoses, bacteria, serotypes

**To the Editor:** Several wildlife-associated zoonotic agents have played a major role in the emergence of diseases in humans (*1*). However, diseases can also emerge in wildlife as a result of human activities, such as contamination of the marine environment and its fauna by the disposal of nontreated human sewage. *Salmonella enterica* is among the agents identified as causing infection in various marine birds and mammals, including pinnipeds, from different geographic regions (*2**–**4*).

The objective of our study was to determine whether *S. enterica* infection occurs in pinnipeds from the Chilean coast. During August–December 2010, we obtained samples from 13 South American sea lions (*Otaria flavescens*) that the sanitary authority found malnourished and stranded at the northern Chilean beaches of Antofagasta (23°40′S; 70°24′W) and Los Vilos (31°54′S; 71°30′W) (Table). The pinnipeds showed no clinical signs or symptoms of disease; however, rectal swab samples were obtained during their stay for rehabilitation at the Buin Marino facilities (Santiago, Chile). After the animals recovered, they were released to their original habitat.

**Table:** Characteristics of South American sea lions (*Otaria flavescens*) tested for infection with *Salmonella* spp., Chilean coast, August–December 2010*

Identification no.	Sex	Age†	Source, city in Chile	*S. enterica* serotype isolated
p070810	F	Juvenile	Antofagasta	ND
p240810	F	Juvenile	Los Vilos	ND
p260810	F	Juvenile	Los Vilos	ND
p090910–1	M	Pup	Antofagasta	Havana
p090910–2	F	Pup	Antofagasta	ND
p140910	M	Pup	Antofagasta	Newport
p011210–1	F	Pup	Antofagasta	ND
p011210–2	F	Pup	Antofagasta	ND
p011210–3	F	Pup	Antofagasta	ND
p011210–4	F	Pup	Antofagasta	ND
p011210–5	F	Pup	Antofagasta	ND
p011210–6	F	Pup	Antofagasta	ND
p011210–7	F	Pup	Antofagasta	ND

*ND, no detection. †Juvenile, animal 1–5 years of age; pup, animal <1 year of age.

The swab samples were placed in Cary-Blair transport medium (COPAN, Murrieta, CA, USA) for shipment to the laboratory (Laboratory of Infectious Diseases, University of Chile, Santiago). To isolate bacteria, we placed the swab samples into 5 mL of buffered peptone water (Difco APT broth; Becton Dickinson, Franklin Lakes, NJ, USA), incubated them for 24 h at 42°C with agitation, and then aliquots of the suspension were transferred into the following media: modified semisolid Rappaport-Vassiliadis basal medium (Oxoid, São Paulo, Brazil) with novobiocin (20 µg/mL), selenite cysteine broth base (Oxoid), and xylose lysine desoxycholate agar (Difco XLD; Becton Dickinson). After the aliquots were incubated at 37°C for 24–48 h, we identified suspected colonies by using biochemical tests and *invA* gene detection by PCR (*5*). Results showed that 2 of the 13 animals were infected with *S. enterica* strains, which were serotyped as *S. enterica* serotype Newport and *S. enterica* serotype Havana (Table), according to the Kauffmann-White scheme (*6*). Testing showed that the strains were susceptible to the following antimicrobial drugs: ampicillin, chloramphenicol, tetracycline, amoxicillin/clavulanic acid, trimethoprim/sulfamethoxazole, cefotaxime, nalidixic acid, nitrofurantoin, ciprofloxacin, ceftazidime, and cefoxitin (*7*).

Our results confirm *S. enterica* infection in pinnipeds from Chile and, more broadly, the South American coast and contrast with previous unsuccessful attempts to detect *Salmonella* spp. in pinnipeds from Valdivia, 2,200 km to the south (*8*). This finding suggests geographic variability in the epidemiology of infection; however, this possibility must be confirmed in additional studies with more samples and additional regions.

*S.* *enterica* is an endemic bacterium in Chile that causes infection in humans and domestic animals. The Chilean sanitary authority includes *S. enterica* infection among the list of notifiable diseases, but surveillance is not conducted for *S. enterica* in wildlife. However, consideration should be given to changing this situation, given a report suggesting *S. enterica* as a priority for active surveillance (*9*). In addition, *S. enterica* serotypes Newport and Havana have been detected in Chile’s human population (*10*), strengthening the necessity for official support for initiatives addressing the need to elucidate the epidemiology of *Salmonella* in aquatic animals.
